# Affective Brain-Computer Music Interfaces—Drivers and Implications

**DOI:** 10.3389/fnhum.2021.711407

**Published:** 2021-06-29

**Authors:** Elisabeth Hildt

**Affiliations:** Center for the Study of Ethics in the Professions, Illinois Institute of Technology, Chicago, IL, United States

**Keywords:** brain-computer interaction, music, direct to consumer, ethics, enhancement, privacy, affective brain-computer interface, neuroessentialism

Brain-computer interfaces (BCIs) allow users to control a computer or other device with their brain activity. While BCI technology has been developed and used primarily in medical contexts, a broad spectrum of non-clinical applications is on the horizon, including fields like concentration management, sleep improvement, music, and painting (Gürkök and Nijholt, 2013; Coates McCall and Wexler, 2020; Saha et al., 2021).

Some BCI applications directly translate brain activity to music performance, offering ways for people with physical disabilities as well as for artists to express their emotions through music (Eaton et al., 2015; Daly et al., 2016; Deuel et al., 2017; Williams and Miranda, 2018).

The focus of this article is on affective BCIs that allow to identify and influence a person's affective states. In addition to providing users with suggestions for the music they like, some affective brain computer music interface (aBCMI) applications aim at modulating the affective states of their users (Daly et al., 2016, 2020; Williams and Miranda, 2018; Ehrlich et al., 2019): Based on determining how listeners respond to certain types of music, music that influences their emotional states can be chosen. These affective BCIs detect correlates of a user's current affective state and attempt to modulate it by generating or selecting music that, for example, serves to increase happiness or reduce stress levels.

While the future development of this type of technology is largely unclear, for future non-clinical aBCMI home applications to be of interest to a broad spectrum of potential users, the technology not only has to prove attractive to a wide audience but also must be ethically sound. Against the background of recent developments in direct-to-consumer (DTC) devices, in what follows, I will discuss driving forces behind aBCMI technology development and social and ethical aspects of the technology, focusing on the perceived role of the brain, mood enhancement, and privacy-related aspects.

## Direct-To-Consumer Devices—Recent Developments

Based on research studies carried out with aBCMIs in complex research environments, several steps have been taken toward developing DTC home applications of aBCMI technology. For example, Kalaganis et al. (2016) developed a consumer BCI that serves to evaluate music in popular on-demand streaming services.

Among the existing wearable devices is the Mico system, a concept model developed in 2013 which allows individualized music choice^1^. Mico is short for “Music inspiration from your subconsciousness,” it consists of headphones and an app for iPhone. According to the developers' homepage, the sensor in the headphone detects brainwaves; the mico app then analyzes the input and matches it to the closest “neural pattern” in the database to identify the user's “neural group.” Based on this categorization, the system selects and plays music from a music database in line with the user's status.^1^

The wearable EEG headset developed by Imec seeks to measure and influence the emotions of the person wearing the headset: According to the company^2^, the system can learn the musical preferences of the users, compose, and play back music in real time that is in line with their preferences, and influence their emotions to achieve the desired emotional state.

The portable and wearable EEG device called Crown™ developed by Neurosity is advertised as being able to boost productivity^3^. According to the company's homepage, the system detects brainwaves and plays music from Spotify that helps users to focus. The increase in productivity brought about by the device seems to be quite small, however (Koetsier, 2021, cf. video interview).

While these are interesting developments, no DTC devices display research-grade results. It is currently unclear whether any of these devices can fulfill what they promise (see Coates McCall and Wexler, 2020). In view of this, it will be important that manufacturers and companies avoid exaggerated claims about the devices' presumed capabilities, not only for reasons of decency in selling the product but also to avoid users generating inadequate beliefs about their affective states. Unrealistic consumer expectations may result in a hype followed by disappointment (van Lente et al., 2013).

## The Role of the Brain

The aBCMI technology promises to provide an individualized music experience, based on the users' brain data. This individualized approach seeks to allow for easier access to music in line with an individual's preferences and needs. As advertised, the mico DTC device “frees the user from having to select songs and artists and allows users to encounter new music just by wearing the device^4^.”

While this certainly alleviates the users from the burden of complex choices, it also narrows down individual decision-making. Instead of individual choice, users listen to what the technology suggests. While a user is free to accept or reject the choice made by the system, the system clearly influences the music someone listens to, be it through the music database provided or through the headset and data processing.

Generally speaking, aBCMI use raises awareness of the role of the brain and the relevance of brain-related data. Brain data serves to specify user affective states, categorize users into user types, and define the beginning and end points of affect-modulating interventions.

As Duncan Williams and Eduardo Miranda write in the context of music therapy (Williams and Miranda, 2018, p. 201):

“The theoretical advantage of this approach over conventional music therapy approaches is that the BCMI is able to directly monitor the users' emotional state via physiological indices of emotion, which have the potential to be more robust and objective measures of emotion than user reports or even the expertise of the music therapist.”

Accordingly, at least with research grade aBCMIs in the context of music therapy, the technology is expected to provide a more direct, more reliable measure of emotion than subjective reports, be it user self-assessments or reports by third persons.

It is worth mentioning that this position touches on tricky longstanding philosophical questions on the epistemic authority of claims about sensations (Baier, 1962): Who or what can be more reliable—a technology that depends on brain recordings or a person's first-person perspective that is based on introspection?

Instead of characterizing a person as being in a certain mood or liking certain music by watching their overt behavior or relying on their introspective reports, with this technology-based approach it is brainwaves that help to find out about a person's emotions, what music they like, and how their mood is influenced by music. By focusing not on a person and their behavior but on brain-related data, the role of the brain is stressed.

While it may be argued that this is just the mechanism upon which aBCMIs rely, when talking or writing about aBCMIs, it is important to avoid neuro-realist interpretations that consider brain activation patterns as the ultimate proof that a phenomenon is real and objective, as well as neuro-essentialist interpretations that see the brain as the self-defining essence of a person (see Racine et al., 2010; Reiner, 2011).

Phrases like “Mico-Music inspiration from your subconsciousness” or “This EEG headset can tell what music your brain likes” (Shankland, 2018), or the characterization of BCI-based music experiments as bringing “a new meaning to ‘straight off the dome”' (Chung, 2017), all consider the brain to be the central actor, a substitute for the person.

## Mood Enhancement and Brain Augmentation

Even though the headsets of DTC devices resemble traditional headsets, and aBCMI-mediated music consumption shares considerable similarities with other forms of music consumption and automated playlist selection technologies, there is a significant difference in that aBCMIs aim at positively influencing affective states.

While the term enhancement—and the distinction between treatments and enhancements—has been used as a boundary concept to characterize interventions as outside the realm of medicine (Frankford, 1998; Juengst, 1998), in more general terms, enhancements are procedures to augment a person's physical or mental capabilities (Lebedev et al., 2018; Coates McCall and Wexler, 2020).

A number of enhancement approaches based on pharmaceuticals or neurotechnology including BCIs have been described in recent years (Zehr, 2015; Cinel et al., 2019). Non-clinical aBCMI home applications that seek to make healthy users feel happier or more relaxed can be considered mood enhancement technologies, devices that aim at helping users to get focused and increase their productivity can be seen in the context of cognitive enhancement.

Whereas pharmacological mood enhancers such as Prozac or other selective serotonin reuptake inhibitors (SSRI) are prescription drugs used for purposes other than originally intended and bear the clear risk of negative systemic side effects (Schermer, 2015), the situation is different for aBCMIs. It is enhancement purposes that current DTC devices are developed for. The aBCMI technology is based on the individual user's brain data, and consists of an individualized approach which in principle could allow each user to navigate very fine grained affect modulations. While negative effects of the technology cannot be excluded, an EEG-based headset can be disconnected easily at any time.

Whereas positive effects of DTC aBCMI devices have not been proven yet, on the one hand, it may be argued that the technology seeks to enable authenticity and increase autonomy in that it allows users to maintain, influence, or attain their desired affective states. On the other hand, outsourcing the responsibility for regulating and controlling one's affective states can be seen as essentially inauthentic and as limiting one's capabilities and autonomy in that it increases dependence on technology (see Steinert and Friedrich, 2020).

## Privacy and Data Protection

In general, issues related to privacy play an important role whenever brain-related data is being collected and stored. In the context of neurotechnologies, several authors have stressed security and privacy risks and argued toward a right to mental privacy and a right to mental integrity (Ienca and Andorno, 2017; Ienca et al., 2018; Lavazza, 2018).

There are a number of privacy aspects to consider around aBCMIs, even though it seems questionable whether current aBCMIs can reveal any detailed information on a person's thoughts or preferences (Coates McCall and Wexler, 2020). In aBCMIs, each EEG recording gives some indication of a person's affective state at a certain point in time. Over a longer period, this will add up to a relatively detailed profile of a user's affective states. Data protection and privacy require that the individual user be in control of what is recorded, how the recordings are stored, and what is revealed and shared by the system about data analysis and classification results.

While the future of DTC aBCMIs is uncertain, with wearable, smartphone-compatible devices, privacy issues can be expected to become even more central. In general, brain-related data processed and stored on a smartphone connected to the internet is susceptible to being the subject of a multitude of data collection and sharing pathways. This could potentially include future individualized nudging or neuromarketing approaches (Ienca et al., 2018; Steinert and Friedrich, 2020).

Data protection measures (such as encryption of brain activity) will have to be established in order to prevent unauthorized access, sharing and use of brain-related data (Hernandez, 2016; Koetsier, 2021).

## Conclusion

Despite these first steps toward developing non-clinical aBCMI home applications, the future of direct-to-consumer aBCMI technology is uncertain. A broad spectrum of challenges will have to be addressed, including electrode development and placement, user comfort, validity, reliability, privacy, and costs. To be attractive, the technology will have to provide some clear benefits to its users, be it regarding human performance, well-being, or leisure activities. At the same time, it will be important to avoid brain-centric interpretations and overreliance on technologically mediated affect management.

## Author Contributions

The author confirms being the sole contributor of this work and has approved it for publication.

## Conflict of Interest

The author declares that the research was conducted in the absence of any commercial or financial relationships that could be construed as a potential conflict of interest.

## Footnotes

^1^https://neurowear.com/mico/

^2^https://www.imec-int.com/en/articles/imec-and-holst-centre-introduce-eeg-headset-for-emotion-detection

^3^https://neurosity.co/

^4^https://neurowear.com/mico/
